# Resistance exercise dose effects on muscle morphology, muscle function and quality of life in advanced-stage ovarian cancer survivors

**DOI:** 10.1007/s00520-025-09401-0

**Published:** 2025-04-10

**Authors:** Christelle Schofield, Marit Mol, Dennis R. Taaffe, Laurien M. Buffart, Pedro Lopez, Robert U. Newton, Daniel A. Galvão, Paul A. Cohen, Carolyn J. Peddle-McIntyre

**Affiliations:** 1 Exercise Medicine Research Institute, Edith Cowan University, 270 Joondalup Drive, Joondalup, Western Australia 6027 Australia; 2 School of Medical and Health Sciences, Edith Cowan University, Joondalup, Western Australia Australia; 3 Department of Medical BioSciences, Radboud University Medical Center, Nijmegen, The Netherlands; 4 Grupo de Pesquisa Em Exercício Para Populações Clínicas (GPCLIN), Universidade de Caxias Do Sul, Caxias Do Sul, Rio Grande Do Sul Brazil; 5 Pleural Medicine Unit, Institute for Respiratory Health, Nedlands, Western Australia Australia; 6 Western Australian Gynaecological Cancer Service, King Edward Memorial Hospital, Perth, Western Australia Australia; 7 Division of Obstetrics and Gynaecology, Medical School, University of Western Australia, Crawley, Western Australia Australia

**Keywords:** Advanced ovarian cancer, Resistance exercise, Exercise dose, Muscle morphology

## Abstract

**Aim:**

Advanced-stage ovarian cancer survivors often have compromised muscle morphology (muscle mass and density), muscle function (muscle strength and physical function), and health-related quality of life (HRQoL). We recently reported improvements in these outcomes following resistance training. Information on the resistance exercise dose required to improve health-related outcomes is still lacking in this cancer group. Here we examined the exercise dose delivered and the effect of the delivered dose on changes in outcomes of interest.

**Methods:**

Twelve women with stage III or IV ovarian cancer completed a 12-week supervised resistance exercise intervention. Exercise metrics included compliance (exercise dose completed), dose modifications (sessions modified) and tolerance (rating of perceived exertion; RPE). Participants were allocated to lower (< 63%) or higher (> 63%) exercise compliance based on median split. Differences in change to muscle morphology, muscle function and HRQoL between compliance groups were investigated.

**Results:**

Median compliance and session RPE were 63.0% and 13 (somewhat hard), respectively. Dose reductions occurred in 92.7% of sessions. Both groups experienced improvements in muscle morphology and function. Higher compliance was associated with greater improvements in whole body lean mass (+ 1.3 kg vs. + 0.5 kg) and lower body strength (+ 50 kg vs. + 13 kg). Only the lower compliance group experienced a clinically significant improvement in 400-m walk time (-48.4 s vs. -9.4 s). Both groups experienced clinically meaningful improvements in social and cognitive functioning.

**Conclusion:**

Relatively lower doses of resistance exercise may benefit advanced-stage ovarian cancer survivors. Exercise programs may need to be flexible and individualized to fit the needs of this cancer group.

## Introduction

Ovarian cancer is the eighth most common cancer in women worldwide, with approximately 324,000 diagnoses in 2022 [1]. Due to a lack of effective screening and nonspecific symptoms, seven out of ten cases are diagnosed at an advanced stage (i.e., stage III or IV) [2]. Consequently, recurrence rates are high [3], and the 5-year survival rate is less than 50% [4]. First-line treatment for advanced-stage ovarian cancer includes primary surgery followed by chemotherapy, or neoadjuvant chemotherapy (NACT) followed by interval surgery [5]. As a result of the high disease and treatment burden, women often experience a range of disease- and treatment-related symptoms and side effects such as muscle loss, fatigue, peripheral neuropathy, insomnia, mood disorders, pelvic floor symptoms and reduced health-related quality of life (HRQoL) [6–8].

It is well-recognized that exercise is beneficial for many cancer populations [9]. However, in ovarian cancer, exercise research is limited, with studies consisting mostly of home-based aerobic or multi-modal interventions [10, 11]. Existing evidence indicates that exercise in this patient group is safe and feasible [10], with women experiencing reduced treatment-related side effects, such as fatigue [10, 12] and peripheral neuropathy [13], and improved muscle strength, physical function and HRQoL [10]. Little is known about the impact of resistance exercise specifically in ovarian cancer [14, 15]. We recently reported improvements in muscle morphology (i.e., muscle mass and density), muscle function (i.e., muscle strength and physical function) and some HRQoL domains in advanced-stage ovarian cancer survivors who completed a 12-week supervised resistance exercise intervention [14], highlighting the potential importance of resistance exercise in ovarian cancer supportive care. While attendance of exercise sessions was high (92%), further exercise metrics such as exercise dose, compliance and tolerance were not explored. Emerging research in other cancer groups suggests that relatively low doses of resistance exercise may be sufficient to elicit beneficial improvements in health-related outcomes [16, 17].

Women diagnosed with advanced-stage ovarian cancer are deconditioned [18, 19] and most do not meet the public health physical activity guidelines [10]. Therefore, modest doses of exercise could have a beneficial effect on health-related outcomes [5]. To our knowledge, the relationship between exercise dose and health-related outcomes has not been investigated in this patient group. Therefore, the aim of this secondary analysis was to determine the dose of resistance exercise delivered compared to the dose planned, and compare the effect of exercise dose on muscle morphology, muscle function and HRQoL.

## Methods

### Setting and participants

This is a secondary analysis of a prospective single-arm study that investigated the effects of supervised resistance exercise, delivered in an exercise clinic or by telehealth, in women with advanced-stage ovarian cancer (stage III or IV) who had completed first-line treatment within the previous four to twelve weeks [14]. Only outcomes that displayed statistically significant and/or clinically meaningful change from pre- to post-intervention were examined in this secondary analysis (i.e., muscle mass and density, muscle strength, physical function, global health/QoL, cognitive and social functioning, fatigue, and dyspnea). Further, only women who participated in the in-clinic intervention were included due to differences in intensity of resistance exercise options available for telehealth.

The study was conducted at the Exercise Medicine Research Institute, Edith Cowan University (ECU), in Perth, Western Australia. Participants were recruited from two major hospitals in Perth, the Sir Charles Gairdner Hospital and St John of God Subiaco Hospital. Women were excluded if they were younger than 18 years, unable to communicate in English, participated in resistance exercise at least twice/week during the last three months, did not receive approval from their treating medical oncologist or general practitioner, were on ≥ 10 mg/day of prednisolone or similar steroid medication in the last four weeks before the intervention, received experimental anti-cancer therapy within eight weeks of starting the study, in need of a blood transfusion within the first eight weeks of starting a PARP inhibitor, or had expected or confirmed bone metastases, or any illness or disorder that could put them at risk during the exercise intervention [14].

### Exercise intervention

The exercise intervention is described in detail elsewhere [14]. In brief, supervised resistance exercise was undertaken twice weekly, on non-consecutive days for 12 weeks. Exercise sessions were performed in an exercise clinic with one-on-one supervision by an accredited exercise physiologist and generally consisted of a 5-min warm-up, 40–50 min of resistance exercise, and five minutes of stretching. Participants were prescribed two to three sets of eight to 12 repetitions of eight exercises at a resistance that was perceived as moderate to hard based on each participant’s individual experience [20]. Exercise intensity gradually increased according to the protocol whenever possible, based on participant’s feedback on perceived exertion and symptoms such as fatigue, pain or neuropathic or musculoskeletal discomfort. Exercises were adapted or replaced by relatively easier exercises if participants were unable to perform the initially prescribed exercises. For example, chair sit-to-stands were performed if participants could not execute a leg press. Additional rehabilitation exercises were included if participants could not execute specific movement patterns due to treatment-related side effects or pre-existing comorbid conditions. After each supervised session, participants rated their perceived exertion according to the Borg Rating of Perceived Exertion (RPE) 6–20 scale [21]. All outcomes were assessed pre- and post-intervention (week 13 or 14).

### Outcome measures

#### Exercise dose

Participants’ training logs were examined for attendance, exercise dose, compliance, exercise load, and tolerance. Reasons for variation to the prescribed exercise program were also examined. Attendance was quantified as the percentage of the total planned sessions (i.e., 24) attended. Exercise dose was calculated as the total volume of exercise completed; a product of the number of sessions, exercises, sets, and repetitions [22]. Compliance was quantified as a percentage of the initially prescribed exercise dose completed. Exercise load was quantified as the product of the number of sessions, sets, repetitions and external load [22, 23]. Tolerance was determined by the RPE of each session. Any change to the prescribed exercise program regarding volume was defined as dose modifications. This included any exercise sessions requiring dose reduction or dose escalation, based on a change in the number of exercises, sets, or repetitions [22, 23].

#### Muscle morphology

Whole-body lean mass (kg) was assessed by dual-energy x-ray absorptiometry (DXA, Hologic Discovery A, Waltman, MA, USA). Appendicular lean muscle (ALM, kg) was obtained from the sum of upper-limb and lower-limb bone-free lean mass [24]. To establish the number of participants with low muscle mass, we used the ALM index cutoff value of 5.45 kg/m^2^ recommended by Baumgartner et al. [25]. Muscle density (mg/cm^3^) of the lower leg was measured by peripheral quantitative computed tomography (pQCT, XCT-3000, Stratec, Pforzheim, Germany) and was obtained from the 66% slice of tibial length [26].

#### Muscle function

Upper and lower body muscle strength were examined using the one-repetition maximum (1RM) test for chest press and the five-repetition maximum (5RM) test for leg press [27], respectively. Relative muscle strength was calculated as the absolute strength divided by body weight. Physical function was determined using the 400-m walk test (a measure of cardiorespiratory fitness and walking endurance) [28] and the Timed Up and Go (TUG) test (a measure of mobility and dynamic balance) [29]. A change of 20 to 30 s in the 400-m walk test is considered minimally clinically significant, while a change of 50 to 60 s is considered substantially clinically significant [30].

#### Quality of life

HRQoL was measured with the European Organisation for Research and Treatment of Cancer Quality of Life Questionnaire Core 30 (EORTC QLQ-C30) version 3.0 [31]. The EORTC QLQ-C30 consists of five functional scales (physical, role, cognitive, emotional, and social functioning), nine symptom scales (fatigue, pain, nausea, vomiting, dyspnea, insomnia, appetite loss, constipation, diarrhea, and financial difficulties), and a global health/QoL scale [31]. Scores range from 0 to 100. Higher scores for functional and global health/QoL scales represent better function and global health/QoL, whereas higher scores for the symptom scales represent a higher symptom burden. A difference/change of 10 points or more is considered clinically meaningful [32].

### Statistical analysis

Data were analyzed with SPSS Statistics Version 27 (IBM Corp) and Microsoft Excel (Version 16.66.1). We used a median split to allocate participants into a lower and a higher exercise compliance group. Descriptive statistics were used to analyze exercise metrics and compare pre- to post-intervention changes in muscle morphology, muscle function and HRQoL between compliance groups. All values are reported as median and range, or number and percentage.

## Results

### Participant characteristics

Twelve of the 15 participants of the original study completed the exercise intervention in the exercise clinic and were therefore included in this analysis. Participants had a median age of 63.5 (range: 33.3 to 72.4) years and median BMI of 25.9 (range: 18.0 to 46.1) kg/m^2^. Most participants were partnered (75.0%), completed secondary school (58.3%), and received NACT and interval surgery (66.7%). Median compliance with the resistance exercise intervention was 63.0%.

Compared to the higher compliance group, the lower compliance group was older (67.7 vs 56.4 years), had a lower BMI (23.8 vs 26.6 kg/m^2^) and was more likely to have received NACT and interval surgery (83.3% vs 50.0%) (Table 1). At baseline, the lower compliance group had lower values of whole-body and appendicular lean mass than the higher compliance group. Three participants in the lower compliance group (50%) compared to none in the higher compliance group had low muscle mass (ALM index ≤ 5.45 kg/m^2^) [25]. The lower compliance group had lower upper and lower body strength and slower 400-m walk and TUG time, but reported better (by > 10 points) social and cognitive functioning and global health/QoL, and less (by > 10 points) fatigue and dyspnea. Five lower compliance participants versus two higher compliance participants required rehabilitation exercises (Table 2).

**Table 1:** Demographic characteristics and pre-intervention outcome measures per exercise compliance group

Variable	Lower compliance group (< 63%) (*n* = 6)	Higher compliance group (> 63%) (*n* = 6)
**Age (years)**	67.7 (46.0 to 71.3)	56.4 (33.3 to 72.4)
**BMI (kg/m**^**2**^**)**	23.8 (18.0 to 28.1)	26.6 (22.8 to 46.1)
**Treatment received**		
Primary surgery and adjuvant chemotherapy	1 (16.7%)	3 (50.0%)
Neoadjuvant chemotherapy and interval surgery	5 (83.3%)	3 (50.0%)
**Relationship status**		
Partnered	4 (66.7%)	5 (83.3%)
Not partnered	2 (33.3%)	1 (16.7%)
**Educational attainment**		
Completed secondary school	5 (83.3%)	2 (33.3%)
Postsecondary certificate/diploma	1 (16.7%)	1 (16.7%)
University degree	0 (0.0%)	3 (50.0%)
**Employment status**		
Currently working part-time	1 (16.7%)	2 (33.3%)
Currently not working	1 (16.7%)	2 (33.3%)
Retired	4 (66.7%)	2 (33.3%)
**Smoking status**		
Non-smoker	3 (50.0%)	3 (50.0%)
Past smoker	3 (50.0%)	3 (50.0%)
**Comorbidities**		
0	2 (33.3%)	2 (33.3%)
1	2 (33.3%)	2 (33.3%)
≥ 2	2 (33.3%)	2 (33.3%)
**Muscle morphology**		
Whole-body lean mass, kg	36.5 (29.4 to 45.7)	39.9 (34.3 to 53.2)
Appendicular lean mass, kg	14.3 (10.9 to 19.0)	15.9 (12.9 to 21.8)
Appendicular lean mass index ≤ 5.45 kg/m^2^	3 (50.0%)	0 (0%)
Muscle density, mg/cm^3^	73.7 (62.8 to 75.6)	73.2 (71.4 to 73.6)
**Muscle function**		
***Absolute strength***		
1 RM chest press, kg	11.3 (5.0 to 16.0)	22.5 (10.0 to 22.5)
5 RM leg press, kg	36.0 (17.0 to 60.0)^§^	70.0 (70.0 to 80.0)^**Ф**^
***Relative strength***		
1 RM chest press, kg/kg body mass	0.16 (0.11 to 0.22)	0.24 (0.17 to 0.36)*
5 RM leg press, kg/kg body mass	0.78 (0.34 to 0.82)^§^	1.05 (0.66 to 1.20)^**Ф**^
***Physical function***		
400-m walk, seconds	364.0 (249.7 to 540.0)	247.0 (238.3 to 337.4)
Timed-up-and-go test, seconds	9.3 (7.2 to 11.4)	6.6 (5.44 to 8.16)
**Health-related QoL**		
***Functional scales***		
Cognitive functioning	83.3 (66.7 to 83.3)^	66.7 (33.3 to 83.3)
Social functioning	66.7 (16.7 to 83.3)^	33.3 (16.7 to 100.0)
***Symptom scales***		
Dyspnea	0.0 (0.0 to 33.3)^	33.3 (0.0 to 66.7)
Fatigue	33.3 (0.0 to 66.7)^	55.6 (0.0 to 100.0)
*** Global health/QoL***	66.7 (58.3 to 83.3)^**^**^	50.0 (33.3 to 83.3)

Values are presented as median (range) or n (%)

Abbreviations: BMI, body mass index; RM, repetition maximum; QoL, quality of life

Whole body and appendicular lean mass were measured by dual-energy X-ray absorptiometry

Muscle density was measured by peripheral quantitative computed tomography

^*^Leg press n = 3 as 3 participants were unable to complete the test

^§^Leg press n = 5 as 1 participant was unable to complete the test

^**Ф**^Chest press n = 5 as 1 participant was unable to complete the test

^^^Quality of life responses n = 5 as 1 participant did not complete questionnaires

**Table 2:** Rehabilitation needs of participants

	Lower compliance group (< 63%)** (*n* = 6)**	Higher compliance group (> 63%)** (*n* = 6)**
**Rehabilitation exercises performed**		
Yes	*n* = 5 (83.3%)	*n* = 2 (33.3%)
No	*n* = 1 (16.6%)	*n* = 4 (66.6%)
**Reasons for rehabilitation exercises**		
Pre-existing musculoskeletal issues	*n* = 4 (66.7%)	*n* = 2 (33.3%)
Treatment-related peripheral neuropathy	*n* = 2 (33.3%)	*n* = 0 (0.0%)
Neurological issues not related to cancer	*n* = 1 (16.7%)	*n* = 0 (0.0%)

Musculoskeletal issues were related to previous shoulder (*n* = 4), ankle (*n* = 1) and knee (*n* = 2) conditions/injuries

Neurological issues not related to cancer were due to recently diagnosed Parkinson’s disease (*n* = 1)

### Exercise metrics

Exercise metrics are presented in Table 3. Median attendance throughout the intervention was 87.5% (range: 75.0 to 100.0), with participants attending 259 of 288 scheduled training sessions. Three participants (25%) attended all 24 sessions. Nine participants (75%) missed a total of 29 sessions with two participants missing one session, three missing three, three missing four and one missing six sessions. Although the total exercise volume prescribed was 5,568 repetitions, the median total volume completed during the exercise program was 3,486 (range: 2,227 to 4,400) repetitions/participant and 166 (range: 111 to 186) repetitions/completed session. This resulted in a median exercise compliance of 63.0 (range: 40.2 to 81.0) %. The median total exercise load completed/participant was 38,914 (range: 7,687 to 71,660) kg with a median load of 1,814 (range: 384 to 2,986) kg/completed session. The median total exercise load and load/session completed by higher compliance participants were nearly three times higher than that completed by lower compliance participants. Regarding exercise tolerance, the median RPE of all sessions was 13.2 (range, 11.3 to 16.2), which corresponds to exercising at a moderate intensity level. Participants rated most sessions with an RPE of 12 to 13, corresponding to ‘somewhat hard’ (n = 136 sessions; 52.5%), or of 14 to 15, corresponding to ‘hard’ (n = 75 sessions; 29.0%).

**Table 3:** Exercise metrics for all participants and per exercise compliance group

Exercise metrics	All Participants (*n* = 12)	Lower compliance group (< 63%) (*n* = 6)	Higher compliance group (> 63%) (*n* = 6)
Attendance (%)	87.5 (75.0 to 100.0)	83.3 (75.0 to 100.0)	95.8 (87.5 to 100.0)
Total exercise dose (repetitions)	3,486 (2,227 to 4,400)	3,016 (2,227 to 3,439)	3,791 (3,532 to 4,400)
Exercise dose per session (repetitions)	166 (111 to 186)	134 (111 to 186)	173 (147 to 183)
Total exercise compliance (%)	63.0 (40.2 to 81.0)	54.8 (40.2 to 62.2)	69.9 (63.8 to 81.0)
Total exercise load (kg)	38,914 (7,687 to 71,660)	19,949 (7,687 to 37,321)	55,936 (40,508 to 71,660)
Exercise load per session (kg)	1,814 (384 to 2,986)	888 (384 to 1,866)	2,498 (1,761 to 2,986)
Exercise tolerance (RPE)	13.2 (11.3 to 16.2)	12.6 (11.3 to 16.2)	13.5 (12.3 to 14.3)

Values are presented as median (range)

Abbreviation: RPE, Rating of Perceived Exertion

Exercise dose = total volume of exercise completed (i.e., product of the number of sessions, exercises, sets and repetitions)

Exercise load = product of the number of sessions, sets, repetitions and external load

**Fig. 1:**
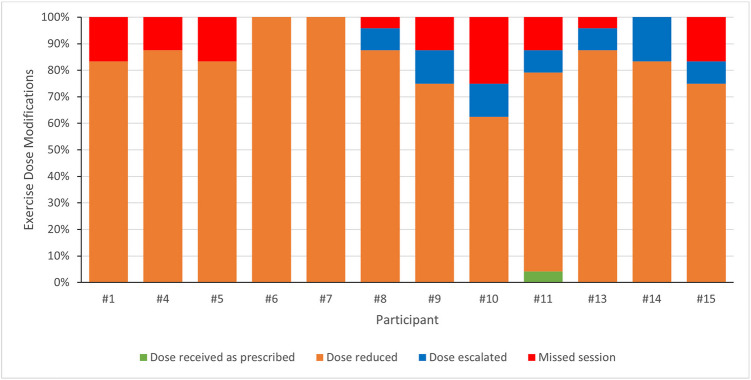
Exercise dose modifications per participant

Exercise dose modifications were applied during 258 of 259 (99.6%) of the completed sessions, with only one session completed as prescribed (Fig. 1). All participants had their dose reduced during the intervention (n = 240; 92.7%), with a median of 20 (range: 15 to 24) sessions/participant at a reduced exercise dose. In most cases (32%, 77 of 240 sessions) exercise dose was reduced by decreasing the number of exercises, repetitions, and sets. Exercise dose was reduced during the exercise program with consideration of pre-existing musculoskeletal and neurological conditions and treatment-related side effects. Seven participants (58.3%) had their exercise dose escalated sometime during the intervention (n = 18 sessions; 6.9%), with a median of two (range: 0 to 4) sessions/participant at an increased exercise dose. In most cases (61%, 11 of 18 sessions) exercise dose was escalated by increasing the number of repetitions.

### Dose modifications

All participants were initially prescribed the same exercise dose, defined as total volume of planned exercise; a product of the number of planned sessions, exercises, sets, and repetitions. It became evident either during the physical assessment pre-intervention, or during the first supervised exercise session that dose modifications were required for some participants. Dose modifications were made at the discretion of the supervising exercise physiologist. The most common modification at the start of the exercise program involved eliminating one or more exercises from the daily planned session, as participants felt too fatigued to complete the entire planned session. Throughout the 12-week period, session-by-session dose modifications were made when required based on participants’ ratings of perceived exertion and self-reported symptoms including fatigue, neuropathy, and musculoskeletal discomfort or pain.

### Exercise dose effects

**Fig. 2:**
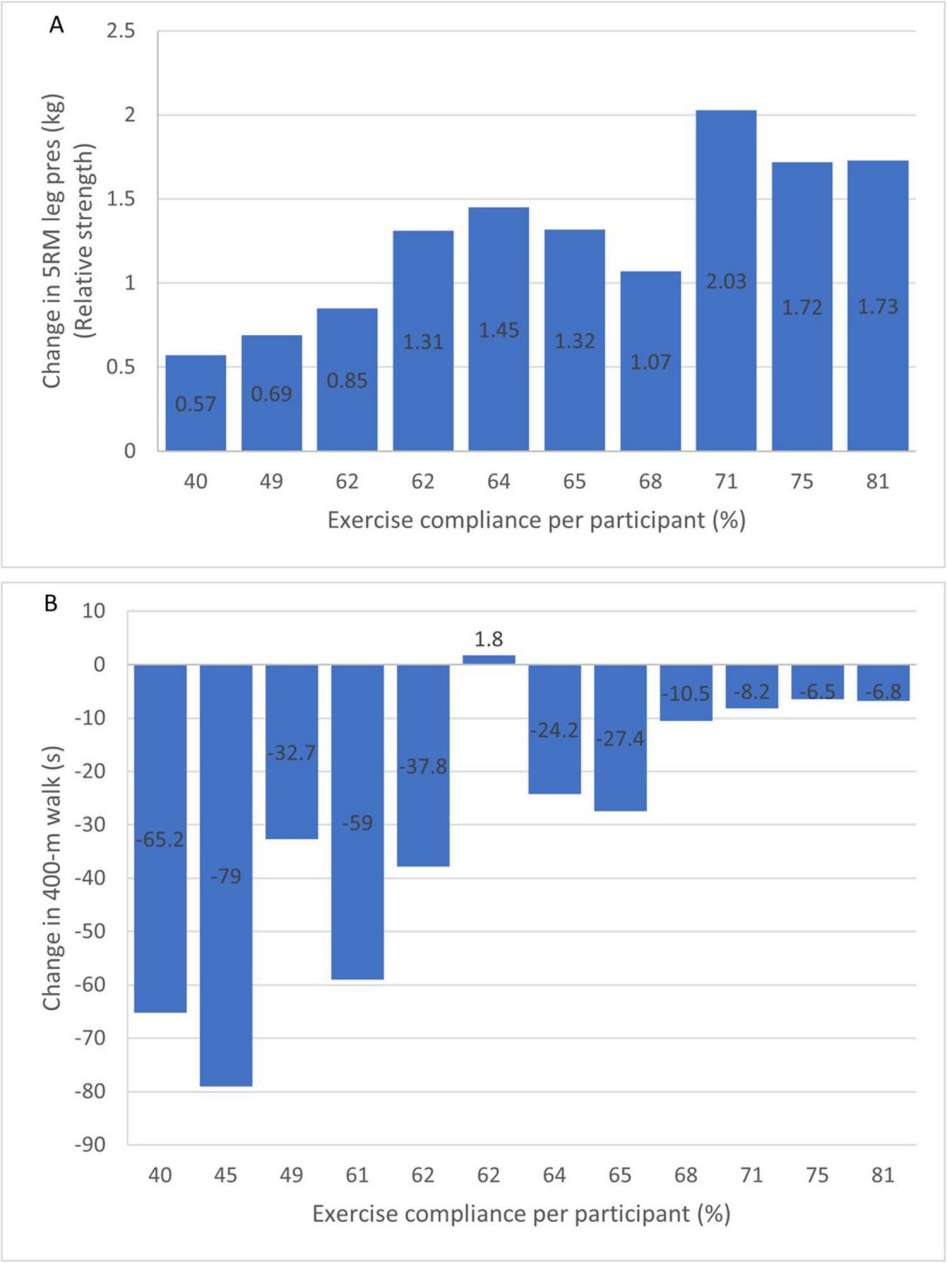
Change in lower body relative strength measured by change in 5 RM leg press (kg) (A) and 400-m walk time (s) (B) relative to exercise compliance (%) per participant

Descriptive statistics of pre- to post-intervention changes in muscle morphology, muscle function and HRQoL for the lower and higher compliance groups are outlined in Table 4. Compared to the lower compliance group, the higher compliance group experienced a greater median change in whole-body lean mass (+ 1.3 kg vs. + 0.5 kg) and ALM (+ 0.9 kg vs. −0.2 kg). For both groups, relative upper body strength increased by 0.1 kg/kg body weight. However, the median increase in absolute and relative lower body strength was 50 (range: 30.0 to 80.0) kg and 0.7 (range: 0.5 to 1.0) kg/kg body weight in the higher compliance group vs. 13 (range: 4.0 to 40.0) kg and 0.2 (range: 0.1 to 0.5) kg/kg body weight in the lower compliance group. The median 400-m walk time was reduced by 9.4 (range: −27.4 to −6.5) seconds in the higher compliance group, compared to 48.4 (range: −79.0 to 1.8) seconds in the lower compliance group, indicating a clinically significant change in the latter group [32]. Change in lower body relative strength and 400-m walk time relative to exercise compliance is presented in Fig. 2.

**Table 4:** Change in muscle morphology, muscle function and health-related quality of life outcomes for lower and higher exercise compliance groups following the intervention

Outcome measures	Lower compliance group (< 63%)		Higher compliance group (> 63%)	
**Muscle morphology**				
Whole-body lean mass, kg	*n* = 6	0.5 (−0.8 to 3.0)	*n* = 6	1.3 (−0.7 to 3.2)
Appendicular lean mass, k	*n* = 6	−0.2 (−0.6 to 2.0)	*n* = 6	0.9 (0.4 to 1.9)
Muscle density, mg/cm^3^	*n* = 6	1.0 (0.3 to 11.5)	*n* = 6	0.7 (−0.8 to 3.4)
**Muscle function**				
***Absolute strength***				
1 RM chest press, kg	*n* = 4	6.0 (2.5 to 12.5)	*n* = 5	8.5 (2.5 to 10.0)
5 RM leg press, kg	*n* = 3	13.0 (4.0 to 40.0)	*n* = 5	50.0 (30.0 to 80.0)
***Relative strength***				
1 RM chest press, kg/kg body mass	*n* = 4	0.1 (0.0 to 0.3)	*n* = 5	0.1 (0.0 to 0.2)
5 RM leg press, kg/kg body mass	*n* = 3	0.2 (0.1 to 0.5)	*n* = 5	0.7 (0.5 to 1.0)
***Physical function***				
400-m walk, seconds	*n* = 6	−48.4 (−79.0 to 1.8)	*n* = 6	−9.4 (−27.4 to −6.5)
Timed-up-and-go test, seconds	*n* = 6	−0.8 (−2.0 to 0.4)	*n* = 6	−0.3 (−0.9 to 0.0)
**Health-related QoL**				
***Functional scales***				
Cognitive functioning	*n* = 5	16.7 (0.0 to 33.3)	*n* = 6	16.7 (0.0 to 33.3)
Social functioning	*n* = 5	16.7 (0.0 to 50.0)	*n* = 6	33.3 (0.0 to 50.0)
***Symptom scales***				
Dyspnea	*n* = 5	0.0 (−33.3 to 0.0)	*n* = 6	−16.7 (−33.3 to 0.0)
Fatigue	*n* = 5	0.0 (−33.3 to 33.3)	*n* = 6	−16.7 (−66.7 to 11.1)
*** Global health/QoL***	*n* = 5	0.0 (−16.7 to 25.00)	*n* = 6	20.8 (−8.3 to 41.6)

Values are presented as median (range)

Abbreviations: RM, repetition maximum; QoL, quality of life

Leg press n = 8 as four participants were unable to perform the leg press pre- and/or post-intervention

Chest press n = 9 as three participants were unable to perform the chest press pre- and/or post-intervention

Questionnaires were completed by 11 participants

Regarding HRQoL, both compliance groups experienced clinically meaningful improvements in cognitive and social functioning, while only the higher compliance group experienced improvements (by > 10 points and thus clinically meaningful) in global health/QoL, fatigue and dyspnea.

## Discussion

We previously reported that a 12-week supervised resistance exercise intervention delivered to advanced-stage ovarian cancer survivors resulted in improvements in muscle morphology, muscle function and certain HRQoL domains. The aims of this secondary analysis were to: 1) determine the dose of resistance exercise delivered compared to the dose planned, and 2) compare the highest and lowest exercise compliance groups to determine the relationship between exercise dose and changes in muscle morphology, muscle function and HRQoL. There were three main findings. First, median resistance exercise compliance was relatively low at 63%. Second, compared to the lower compliance group, the higher compliance group experienced greater increases in muscle mass and lower body strength, while only the lower compliance group experienced a clinically meaningful improvement in 400-m walk time. Third, both compliance groups experienced clinically meaningful improvements in cognitive and social functioning, while only the higher compliance group additionally experienced clinically meaningful improvements in global health/QoL, fatigue and dyspnea.

We found that although attendance of exercise sessions was high at 87.5%, exercise compliance was low at 63%. For all participants, the prescribed exercise dose needed to be reduced in most of the exercise sessions delivered. Our finding of high attendance with low compliance is not consistent with research investigating resistance exercise dose in other cancer populations. Lopez et al. observed a median attendance of 80.6% and a median resistance exercise compliance of 88.5% for men with localized prostate cancer [22], while Fairman and colleagues reported a mean attendance and resistance exercise compliance of 79.5% and 77.4%, respectively, for men with metastatic prostate cancer [23]. In a combined impact and resistance exercise intervention for menopausal breast cancer survivors, mean attendance of supervised sessions was 64% with compliance of 84% [33]. This disparity between findings is likely due to differences in cancer- and treatment-related side effects and demographic characteristics. Our sample of advanced-stage ovarian cancer survivors may have been more deconditioned than participants in the above studies due to a combination of the high burden of advanced disease and primary treatment, side effects of primary and ongoing treatment (five participants received ongoing treatment during the intervention), and early exercise intervention after primary treatment completion. Further, most of our participants had cancer-related side effects or pre-existing comorbidities that necessitated program modification. Nonetheless, while participants completed less than what was prescribed, they still perceived the training program as somewhat hard (median RPE of 13) and beneficial effects were derived. Considering the overall positive effects of the intervention despite the low dose of exercise delivered, the minimal effective exercise dose could be much lower for advanced-stage ovarian cancer survivors than what is currently being prescribed or recommended [14].

Exercise compliance within our sample may have been influenced by demographic factors, pre-intervention physical fitness, and the presence of treatment-related side effects and comorbidities. The median age of the lower compliance group was 11 years older than the higher compliance group. This implies that younger advanced-stage ovarian cancer survivors are more physically fit after primary treatment completion and can therefore tolerate a higher resistance exercise dose. Further, although the lower compliance group had a median pre-intervention BMI considered normal or healthy, half had low muscle mass [25], compared to none of the higher compliance participants. While the association between muscle mass and resistance exercise compliance is unclear, our findings suggest that cancer survivors with higher mass may tolerate higher relative doses of resistance exercise. The lower compliance group also required more rehabilitation exercises for cancer- and non-cancer-related physical issues. These results support the importance of individually tailored exercise prescription, with consideration of baseline physical fitness, presence of cancer-related side effects, and pre-existing comorbidities that necessitate physical rehabilitation [11].

There could be differential effects of resistance exercise dose on muscle morphology and function. In this study, the higher compliance group experienced greater increases in lean mass and lower body strength compared to the lower compliance group. However, the lower compliance group improved more in the 400-m walk. In a previously published study among women with ovarian cancer, Cao et al. [34] observed no significant differences in body composition change between women who met or did not meet a 150 min/week exercise target. However, the exercise intervention involved moderate-intensity aerobic exercise only. The authors did not compare measures of physical function or muscle strength between adherence groups [34]. Lopez et al. [22] reported greater increases in ALM with higher resistance exercise compliance in men with localized prostate cancer, while exercise compliance did not affect physical function and muscle strength. It could be that resistance training influences mobility more for those with the poorest baseline scores. Participants from the lower compliance group in our study had scores below average for the 400-m walk [35] and were notably slower pre-intervention than participants from the higher compliance group. While few studies have investigated the effects of resistance training on muscle function in ovarian cancer, research in other cancer populations shows that resistance exercise alone or in combination with aerobic exercise is more effective in increasing muscle function than aerobic exercise alone [36, 37]. Future exercise oncology studies should investigate the effect of resistance exercise dose on muscle morphology and function to better understand minimal resistance exercise dose requirements across different cancer populations.

Post-intervention, the higher exercise compliance group reported a larger improvement in social functioning than the lower compliance group. Further, the higher compliance group reported clinically meaningful improvements in global health/QoL, dyspnea and fatigue, while the lower compliance group reported no change in these scales. In a study investigating the effect of moderate intensity aerobic exercise in women with ovarian cancer, Zhou et al. [38] observed greater improvements in physical HRQoL for women who met a target of 150 min/week vs those who did not. Those with relatively higher compliance to exercise may experience greater improvements in HRQoL, although studies in other cancer populations investigating the effect of exercise dose on QoL reported mixed results [39, 40]. It is worth noting that, compared to the higher compliance (and younger) group, the lower compliance (and older) group had baseline scores more than 10 points higher for cognitive and social functioning and global health/QoL. The latter group also reported clinically meaningful less fatigue and dyspnea. This finding is consistent with previous research reporting significantly and clinically meaningfully higher cognitive and social functioning and lower fatigue in older versus younger advanced-stage ovarian cancer patients before surgery [41]. While exercise training has been shown to improve HRQoL and decrease fatigue in ovarian cancer [10, 11], future research should investigate the effect of different exercise medicine doses on HRQoL in this cancer population.

This study is the first to report the exercise dose delivered and its effect on different health-related outcomes in ovarian cancer. While previous exercise studies in this cancer population report high adherence rates (defined as average exercise minutes/week, % of participants and/or weeks meeting the exercise target, or session attendance) [10], none had considered compliance. The major strength of this research is that, in addition to attendance, compliance with the exercise program was reported in detail. Further, all training sessions were supervised and reported by the same exercise physiologist, ensuring consistent reporting. However, some limitations are worthy of comment, the main being the small sample size. While the small sample size is a limitation, it reflects the challenges in recruiting women diagnosed with ovarian cancer into exercise studies [11, 42]. A further limitation is the homogeneous nature of our sample in terms of disease and treatment stage, which limits generalisability of findings to women undergoing chemotherapy treatment or women with recurrent disease. Also, the resistance exercise intervention consisted of two supervised, in-clinic training sessions and one unsupervised, home-based session per week. Data from the home-based session was not available and could not be considered in determining the exercise dose, although it could have influenced the outcomes investigated.

## Conclusion

For advanced-stage ovarian cancer survivors participating in a 12-week in-clinic resistance exercise program, attendance was high, but exercise compliance was generally low, with dose modifications in most sessions. Our findings suggest that resistance exercise prescription for this cancer population needs to be flexible and tailored to individual cancer-related health issues and co-morbidities. Despite the relatively low dose of resistance exercise delivered, participants still benefitted, suggesting that, for these women, lower resistance exercise doses could be sufficient to positively influence muscle morphology and function, and HRQoL. More research with larger sample sizes is necessary to investigate resistance exercise compliance and the dose–response relationship in this cancer population.

## Data Availability

No datasets were generated or analysed during the current study.
